# Medical Society of the County of Erie

**Published:** 1908-12

**Authors:** Franklin C. Gram

**Affiliations:** Secretary


### SOCIETY PROCEEDINGS.

### Medical Society of the County of Erie.

Reported by FRANKLIN C. GRAM, M.D., Secretary.

[Received November, 1908.]

A REGULAR meeting of the Medical Society of the County of Erie was held in the rooms of the Society of Natural Sciences, Buffalo Library Building, Monday, October 12, 1908, at 8 p. m. The president, Dr. Edward Clark, in the chair. The minutes of the June meeting, also the minutes of the council meetings of June 29 and August 24 were read and approved.

Dr. McKee, chairman of the membership committee read the list of applicants for membership, as follows: H. W. Cowper, Harry E. Brauer, Leonard Reu, Arthur C. Schaefer. On motion of Dr. Bennett, action was deferred on these applications until the December meeting.

Treasurer Lytle reported certain correspondence which he had with Dr. Woodard, relative to the resignation of the latter. On motion of Dr. Bonnar, the resignation of Dr. Woodward was accepted as effective on the date of its presentation.

Similar action was taken in regard to Dr. Mary C. Hurlburt, who has removed from the county and is now a member of the society where she resides; also regarding Dr. Ira C. Brown, who is in the United States service and absent from the city.

Dr. J. D. Bonnar, chairman of special committee on contagious disease hospital, made a verbal rèport of what the committee had done. A subcommittee, of which Dr. Rochester was chairman, worked with a similar committee from the Academy of Medicine. At a meeting of the entire committee held September 30, 1908, the conclusions of the subcommittee were presented and approved. This report was sent to the Common Council, and the entire matter is likewise up for presentation at a meeting of the Buffalo Academy of Medicine to be held October 14, 1908. This question was so well received throughout the city, that favorable resolutions have been adopted by many civic organisations. The matter will come up before the aldermanic committee on sanitary measures, Thursday, October 29, 1908, and Dr. Bonnar urged everyone to be present.

A letter, from Health Commissioner Wende, relative to the subject, was read, as follows: (This letter appeared in the November issue. 9 v. p. 220).

Dr. DeLancey Rochester, chairman of the subcommittee, briefly stated the work performed and then gave a summary of conclusions and results which were presented to the Common Council, as follows:

*"To the Honorable the Common Council of the City of Buffalo:*

The undersigned, being a committee of the Medical Society of the County of Erie, appointed for the purpose, do hereby petition your honorable body to take immediate steps for the establishment and maintenance of a city hospital for infectious diseases with a capacity for two hundred and fifty (250) patients. The reasons for this request is as follows:

1. During the year from July 1, 1907, to July 1, 1908, there occurred in the city of Buffalo the following numbers of cases of measles, 2,388; scarlet fever, 1,324; diphtheria, 440, and the following number of deaths from measles, 46; scarlet fever, 59; diphtheria, 65; distributed through the different months as per the attached table: There were per 10,000 inhabitants, measles, 60; scarlet fever, 33; diphtheria, 11.

2. The present hospital facilities for receiving such cases are utterly inadequate—the Sisters' hospital being able to accommodate 8 or 10, the Children's hospital, 10, the Erie County hospital, 8. These, we believe are the only institutions which will receive these cases.

3. By the very proper law of the city, all houses in which there are present certain infectious and contagious disease are quarantined for periods varying from two weeks to two months or more, according to the number and character of cases present.

4. When so quarantined, social or business relations are either entirely suspended or greatly diminished. While this is of the greatest value to the health of the city at large, and, therefore, should not in any way be interfered with, it is a hardship upon the individual citizens which will be, to a great extent, obviated by the establishment of such a hospital. For example, if a case of such infectious disease occur in a hotel or boarding house, such hotel or boarding house must be quarantined and placarded: if a domestic employee come down with such disease, the house in which such employee lives must be quarantined and the children of the family kept out of school and the business of the other members of the family interfered with. Some families have the shop and house in the same building. In such cases, the business would be entirely stopped and the source of income interfered with. If such case occur in the family of a woman who goes out to work, her means of livelihood is stopped. If such case occur in a tenement house, the spread of disease is enormous and the impossibility of proper quarantine is evident.

5. The results of treatment of these cases in hospitals are much better than in private practice, both as to diminished death-rate and in the fewer serious sequels.

6 As a result of removal of such cases to hospitals, sources of infection are removed from the people and a fewer number of cases occur in the community, as proven by statistics of those places where such institutions exist—notably in Glasgow, Scotland, London, Eng., Boston, Massachusetts, and New York City. The figures for these places before and after the establishment of these hospitals are at hand if desired.

With these facts before you, we believe it unnecessary to present any prolonged argument as to the necessity of taking steps for the immediate establishment of such a hospital.

John D. Bonnar, DeLancey Rochester,
Herman E. Hayd, Lawrence G. Hanley.

Committee of Erie County Medical Society on the necessity for a City Hospital for Infectious Diseases.

All these reports were received and the thanks of the society extended to the committee for the faithful performance of the duties imposed upon them. On motion of Dr. Rochester, the secretary was requested to read Dr. Wende's letter before the meeting of the Academy of Medicine, October 14 instant.

Dr. Rochester stated that the antispitting ordinance was not being lived up to as it ought to be, and thought it would be wise for the society to appoint a committee to frame certain proper placards regarding the matter. He moved that a committee of three be appointed by the president to take up the matter of framing communications to the public, explaining the deleterious effects of spitting, and empowering the committee to place cards about the city.

Dr. John H. Grant moved an amendment to the motion "that the mottoes, when framed, be presented to the Department of Health so as not to be a charge against the society. The amendment was lost. Dr. Wall moved another amendment "that the committee estimate the cost of such work and submit the entire matter to the next meeting of the society. Dr. Rochester accepted the amendment. The motion, as amended, was then carried.

The President appointed as the committee, Drs. Rochester, Grosvenor, and Wall.

The scientific part of the program was then taken up.

N. G. Russell presented a paper entitled, Cerebral hemorrhage in the newly born; A. G. Bennett read a paper entitled, Some thoughts on sick headache; Helena Kuhlman presented A case of anxiety psychosis; George N. Jack read a paper on Lymph.

All the papers presented were discussed, after which the president thanked those who participated, especially those who had read such excellent papers at this meeting.

Meeting adjourned.

